# Nanogold sol plasmon discattering assay for trace carbendazim in tea coupled aptamer with Au^3+^-glyoxal-carbon dot nanocatalytic reaction

**DOI:** 10.3389/fnut.2023.1122876

**Published:** 2023-03-06

**Authors:** Hongyan Bai, Ran Zhang, Chongning Li, Aihui Liang

**Affiliations:** ^1^School of Public Health, Guilin Medical University, Guilin, China; ^2^Guangxi Key Laboratory of Environmental Pollution Control Theory, Guilin, China

**Keywords:** Fe-doped carbon dot, ferrocene, nanogold indicator reaction, discattering aptamer assay, carbendazim

## Abstract

Carbendazim (CBZ) is a broad-spectrum fungicide, which is toxic to mammals. Therefore, it is very necessary to establish a sensitive detection for food safety. An experiment found that CD_Fe_ exhibited excellent catalysis for the nano-indicator reaction of HAuCl_4_-glyoxal to produce gold nanoparticles (AuNPs) and that the generated AuNPs have a very strong surface-enhanced Raman scattering (SERS) effect at 1613 cm^−1^ in the presence of Victoria blue B molecular probes, and resonance Rayleigh scattering (RRS) signals at 370 nm. The aptamer (Apt) suppressed the catalysis of CD_Fe_ to cause the SERS and RRS signals decreasing. With the addition of CBZ, the specific Apt reaction occurred to restore the catalysis of CD_Fe_, and resulting in a linear increase in the signals of RRS and SERS. As a result, this new nanocatalytic amplification indicator reaction was coupled with a specific Apt reaction of carbendazim (CBZ), to construct a new CD_Fe_ catalytic amplification-aptamer SERS/RRS discattering assay for ultratrace CBZ, which was used to analyze CBZ in tea samples with satisfactory results. In addition, this biosensoring platform can be also used to assay profenofos.

## 1. Introduction

As a new type of carbon nanomaterials, carbon dots (CDs) have attracted great attention due to their excellent water solubility, low toxicity, high stability, and easy modification (1–3). Therefore, the preparation of new CDs always has adaptability. It is a research hotspot in the field of nanomaterials and analytical chemistry. The main methods of the preparation of CDs are the microwave, electrochemical method, laser ablation, and hydrothermal procedures (4–11). In the hydrothermal procedure, a green solvent of water was used, thus adding interest to nanomaterial scientists and analysts. In nanoanalysis, the main application of CDs is fluorescence analysis. Most methods are based on the redox reaction between the CDs fluorescence probe and the analyte, which is a coordination reaction that leads to fluorescence quenching or fluorescence enhancement. Zhao et al. (5) proposed a solvothermal method using corn stalks as raw materials to prepare a CDs-based nanohybrid dual-emission system and used it as a fluorescence sensor to detect Hg^2+^, with a concentration range of 0–40 μM Hg^2+^ and a detection limit (DL) of 9.0 nM. Srinivasan et al. (6) prepared a MoS_2_ nanosheets-DNA-CD complex and used them as fluorescent probes to establish a fluorescence method for the detection of Hg^2+^, with a DL of 1.02 nM. Wang et al. (7) coupled CD with a glyphosate antibody to construct antibody-labeled CD (IgG-CD). The prepared IgG-CD could specifically recognize glyphosate in a concentration range of 0.01–80 μg/ml. Recently, it has been found that the quantum yield of CDs can be improved by doping negative electron heteroatoms such as N, S, and P (8–10). Konar et al. (11) developed nitrogen-doped CDs as a new fluorescent probe for a quantitative determination of l-cysteine and found a linear relationship in the range of 10–210 μM and a DL of 75.6 nM. As a new nanomaterial, iron-doped carbon dots (CD_Fe_) have superior optical stability, cheap raw materials, high-contrast efficiency, and good adaptability. It is expected that nanomaterials will be better used in the application of biomarkers, bioimaging, and drugs. For example, Huang et al. (12) used glutathione (GSH), ethylenediaminetetraacetic acid disodium salt (EDTA), and FeSO_4_ as raw materials and synthesized Fe-CQDs by hydrothermal method, which was suitable for T1-weighted MR/FL bimodal bioimaging and had biocompatibility. Fluorescent CD_Fe_ was prepared by hydrothermal carbonization of methyl thymol blue sodium salt (MTB) and FeCl_3_ as precursors, a fluorescence sensing system based on CD_Fe_ ratio of H_2_O_2_ and glucose was designed by Zhu et al. (13), which quantifies H_2_O_2_ and glucose in the range of 0–133 and 0–300 μm with a DL of 0.47 and 2.5 μM, respectively. To the best of our knowledge, there are few reports on the environmentally friendly preparation and high catalytic activity of CD_Fe_, which was used for nanocatalytic amplification-aptamer surface (Apt)-enhanced Raman scattering (SERS) quantitative analysis of trace carbendazim (CBZ).

Surface-enhanced Raman scattering can sensitively detect the concentration of molecules adsorbed on the nanosurface and provide a wealth of molecular structure information (14). Qi et al. (15) developed a multi-cytosine SERS nanoprobe, which could detect Hg^2+^ quickly, sensitively, and highly selectively and shows a good SERS response in the concentration range of 0.1–1,000 nM. Similarly, a non-pretreatment SERS detection was completed by using a plasma platform functionalized (16). To improve the selectivity and detection range, some selective bioreactions such as Apt and peptide were used in SERS (17–19). Yang et al. (20) established an Apt-SERS sensor for the highly selective detection of thrombin, combining the nanocatalyzed SERS reaction of gold nanoparticles (AuNPs) on HAuCl_4_-H_2_O_2_ with the Apt reaction, which could effectively detect the concentration range of 0.13–53.33 nmol/L Pb^2+^. A stable and cleavable enzyme-coated reductive graphene oxide (rGO)-pdAu probe was prepared by Xu et al. (21) as a signal probe and proposed the visual colorimetric and electrochemiluminescence dual-mode sensing of Pb^2+^. Zong et al. (22) also proposed a colorimetric and SERS dual-mode telomerase activity detection method. On the contrary, resonance Rayleigh scattering (RRS) has the advantages of strong signal, simple operation, good reproducibility, and no introduction of molecular probe reagents (23). Therefore, coupling of SERS and RRS two spectral techniques is of great significance for providing two choices and being of two methods and vantages.

Carbendazim is a broad-spectrum fungicide, which has a wide range of applications and can control many crop diseases caused by fungi. However, its residues can cause liver disease and chromosomal aberration, which are toxic to mammals. Therefore, it is very necessary to establish a sensitive detection method for trace amounts of CBZ in food. At present, the determination methods of CBZ residues in fruits include liquid chromatography, ion chromatography, and liquid chromatography-mass spectrometry/mass spectrometry (24). The most commonly used method was liquid chromatography, since it has strong selectivity. However, these methods have some disadvantages such as expensive equipment, time-consuming, and the pretreatment method requiring complex processes such as liquid–liquid extraction and solid-phase extraction. With the development of new technologies and materials, some new nano-analytical methods for the determination of CBZ have also been reported (25–28). Although these assays have their advantages, their shortcomings cannot be ignored. However, there are few reports on the quantitative determination of CBZ by Apt-SERS method. Therefore, in this article, we avoided the use of organic solvents and developed a hydrothermal procedure to prepare CD_Fe_ with high activity and stability, and the new nanocatalytic indicator reaction of CD_Fe_-HAuCl_4_-glyoxal (GX) was coupled with Apt to establish a new SERS/RRS dimode assay for the detection of CBZ.

## 2. Experimental section

### 2.1. Instrument

UPW-N15UV ultrapure water machine (Shanghai INESA Scientific Instrument Co., Ltd.), KQ3200DB CNC ultrasonic cleaner (Kunshan Ultrasonic Instrument Co., Ltd., ultrasonic electric power 150 W, working frequency 40 kHz), F-7000 Hitachi fluorescence spectrometer (Hitachi, Japan), DXR smart Raman spectrometer (Thermo, USA), transmission electron microscope (FEI Talos 200S, Thermo Fisher Scientific), SXZ-4-10TC Ceramic Fiber Muffle Furnace (Shanghai, China, power 4,000 W, working frequency 50 Hz), K-ALPHA X-ray photoelectron spectrometer (U.S. Thermo Company), HH-S2 electric heating constant temperature water bath (JintanDadi Automation Instrument Factory), high-speed refrigerated centrifuge (Shanghai Lu Xiangyi Centrifuge Instrument Co., Ltd.), FD-1C-50 vacuum Freeze dryer (Hangzhou Jutong Electronics Co., Ltd.), Fourier transform infrared spectrometer (Shanghai, China), and TU-1901 dual-beam ultraviolet-visible spectrophotometer (Beijing, China) were used in the study.

### 2.2. Reagents

Aptamer of CBZ (Apt_CBZ_) with a sequence of GGG CAC ACA ACA ACC GAT GGT CCA GCC ACC CGA ATG ACC AGC CCA CCC GCC ACC CCG CG; aptamer of oxytetracycline (Apt_OTC_) with a sequence of CGA CGC ACA GTC GCT GGT GCG TAC CTG GTT GCC GTT GTG T, and aptamer of profenofos (Apt_PF_) with a sequence of AAG CTT GCT TTA TAG CCT GCA GCG ATT CTT GAT CGG AAA AGG CTG AGA GCT ACG C were bought from Shanghai Shenggong Bioengineering Co., Ltd., Shanghai, China. Carbendazim, oxytetracycline hydrochloride, and profenofos (Shanghai Shenggong Bioengineering Co., Ltd., China); ferrocene (Fer, Tianjin Zhiyuan Chemical Reagent Co., Ltd., China); Hemin (HM, Shanghai Maclean Biochemical Technology Co., Ltd., China); and Victoria blue B (VBB), HAuCl_4_, sodium citrate, glyoxal (GX), and d-galactose (C_6_H_12_O_6_, Shanghai Maclean Biochemical Technology Co., Ltd., China) were used. All reagents used are of analytical grade, and the experimental water is sub-boiling water.

### 2.3. Preparation of Fe carbon quantum dots (CD_Fe_)

We first took an accurately weighed 0.0180 g of ferrocene, then added 0.0180 g of sodium citrate and 30 ml of water, ultrasound for 15 min, transferred it into the autoclave, sealed it, and placed it in the muffle furnace, set the program to 30 min, raised it to 180°C, and kept warm for 2 h. After the reaction was over, the solution was cooled at room temperature with the ice water and diluted to 30 ml with water to obtain a 0.6 mg/ml CD_Fe_ that was calculated as Fer. The results of the catalytic experiment showed that CD_Fe_ had a stronger catalytic effect without dialysis, thus, CD_Fe_ without dialysis was selected as the catalyst in the experiment.

Next, we took an accurately weighed 0.0180 g of Hemin and then added 0.0180 g of sodium citrate and 30 ml water to the digestion tank. A concentration of 0.6 mg/ml of ferrocene carbon dots (CD_HM)_ was prepared by microwave digestion at 180°C for a reaction time of 2 h.

### 2.4. Experimental method

In a 5.0 ml stoppered test tube, a certain concentration of CBZ solution and 180 μl 10 nmol/L Apt were sequentially added, then 120 μl of 0.6 mg/L CD_Fe_ solution was added and bathed in 80°C water for 5 min. Subsequently, 80 μl of 1 mg/ml HAuCl_4_, 180 μl of 0.01 mol/L HCl, and 60 μl of 0.087 mol/L GX were added and diluted to 1.5 ml. After 35 min in a water bath at 80°C, the reaction was terminated with ice water, and the solution was taken in a quartz dish, at volt = 350 V, excited slit = emission slit = 5 nm, and scanned with a fluorescence spectrophotometer to obtain an RRS spectrum. The RRS value at 370 nm was measured and recorded as I_370nm_, and the RRS value without adding CBZ was recorded as a blank value _(_I_370nm)0_. For SERS detection, 100 μl of 10 μmol/L VBB was added and mixed well, the solution was scanned in the Raman spectrum under the conditions of a Raman spectrometer light source power of 1.0 mW and a slit of 25.0 μm and measured the SERS intensity at 1,613 cm^−1^ as I_1,613cm−1_, without CBZ as a blank value (I_1,613cm−1_)_0_, and the value of ΔI_1,613cm−1_=I_1,613cm−1_ *-* (I_1,613cm−1_)_0_ was calculated.

## 3. Results and discussion

### 3.1. Analysis principle

**Figure 1:**
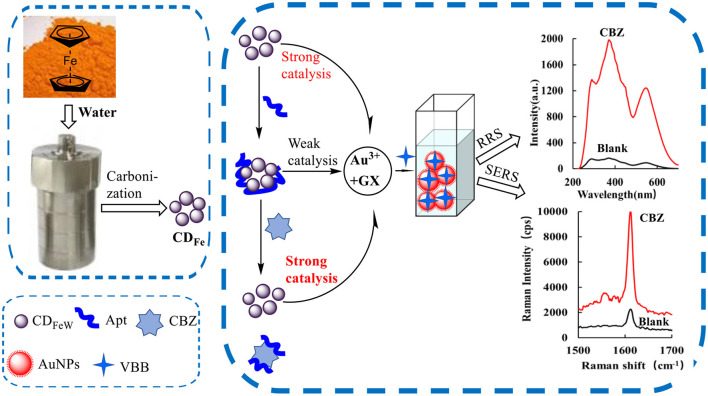
Analytical principle of dimode molecular spectral detection of CBZ based on aptamer-mediated CD_Fe_ catalyzed nanogold reaction.

HAuCl_4_ and GX did not react at room temperature, and it was extremely difficult to react even with underwater bath heating. However, due to its small particle size, when CD was added to the reaction system, the CD_Fe_ could strongly catalyze the reaction of HAuCl_4_ by GX to form AuNPs. The AuNPs generated by the reaction had a strong RRS signal. After adding the VBB probe molecule, AuNPs showed a strong SERS signal. After the addition of Apt, it could be adsorbed on CD_Fe_ to reduce its catalytic activity. The system generated fewer AuNPs, which reduced the SERS/RRS signal of the system. Next, when the target molecule CBZ was added, it would bind specifically to the Apt and then CD_Fe_ would be released so that its catalytic activity would gradually recover. The higher the concentration of the CBZ added, the higher the concentration of CD_Fe_ released and the more the AuNPs generated, and the signal of SERS/RRS of the system was linearly enhanced. Therefore, a new SERS/RRS analysis method for the determination of trace CBZ could be established (Figure 1).

### 3.2. Preparation and molecular spectral characterization of CD_Fe_

#### 3.2.1. Selection of CD_Fe_ preparation conditions

The preparation of CD_Fe_ in the muffle furnace was tested. The conditions for the preparation of CD_Fe_ were tested by the single-factor transformation method. The effects of three factors, namely, Fer dosage, reaction time, and reaction temperature, were investigated, and the prepared CD_Fe_ was used to catalyze the GX-HAuCl_4_ reaction (Supplementary Figures 1–3). The slope k of the linear relationship between the catalyst concentration and the I_370nm_ value of the system was used to evaluate the catalytic effect of the catalyst. That is, the higher the slope, the stronger the catalytic effect. Supplementary Figure 4A shows that as the reaction time increases, the catalytic effect first increases and then decreases. When the reaction time is 120 min, the catalytic activity is the strongest, so the CD_Fe_ reaction time is selected as 120 min. As can be seen from Supplementary Figure 4B, the effect of the catalyst first increases and then decreases with the increase in reaction temperature. As can be seen from Supplementary Figure 4C, with the increasing amount of ferrocene, the catalytic effect first increases and then decreases. When the ferrocene used is 0.018 g, the catalytic activity is the strongest. Therefore, the amount of Fer added is 0.018 g. In summary, the optimal conditions for the preparation of CD_Fe_ by the Fer hydrothermal method are as follows: Fer addition of 0.018 g, adding 30 ml of water, and heating at 180°C for 120 min.

#### 3.2.2. CD_Fe_ fluorescence/RRS/Abs spectrum

The fluorescence spectrum of CD_Fe_ (volt = 500 V, excited slit = emission slit = 10 nm) shows that as the excitation wavelength (λ_EX_) shifts from 300 to 380 nm, there is still no fluorescence intensity (Supplementary Figure 5A). Based on the reported data of carbon quantum dots, it is found that some of them will have fluorescence behavior at the excitation wavelength of 320 nm, thus, when the excitation wavelength is 320 nm, CD_Fe_ shows weak fluorescence behavior (Supplementary Figure 5B). In addition, when the excitation wavelength is 320 nm, the fluorescence peak is roughly distributed at 420 nm. The excitation wavelength of 320 nm was selected to obtain the fluorescence spectra of CD_Fe_ with different concentrations, and the results showed that the fluorescence intensity of CD_Fe_ at 420 nm has a good linear relationship with its concentration within the range of 0–0.128 g/L CD_Fe_ concentration (Supplementary Figure 5B). The fluorescence peak of CD_HM_ is roughly distributed at 445 nm, and there is a good linear relationship between the fluorescence intensity and the concentration in the concentration range of 0–0.096 g/L (Supplementary Figure 5C). The RRS spectrum obtained by the synchronous scanning technique of fluorimeter is a simple and sensitive technique for studying the scattering characteristics of nanoparticles. For the RRS spectrum of CD_Fe_ and CD_HM_ (volt = 350 V, excited slit = emission slit = 10 nm), the RRS intensity at 370 nm showed a linear correlation with the concentration (Supplementary Figures 5D, E), indicating that the particles with larger molecular weight than Fer were formed during the preparation of CD_Fe_. For the absorption spectrum, CD_Fe_ has a wide absorption band at 200–600 nm, and its absorbance gradually increases with an increase in CD_Fe_ concentration, and an absorption peak appears at 250 nm (Supplementary Figure 5F). The absorption band of CD_HM_ is relatively narrower than that of CD_Fe_, but its absorbance also becomes stronger with an increase in CD_HM_ concentration, and its absorption peak appears at 300 nm (Supplementary Figure 5G).

#### 3.2.3. CD_Fe_ infrared and SERS

**Figure 2:**
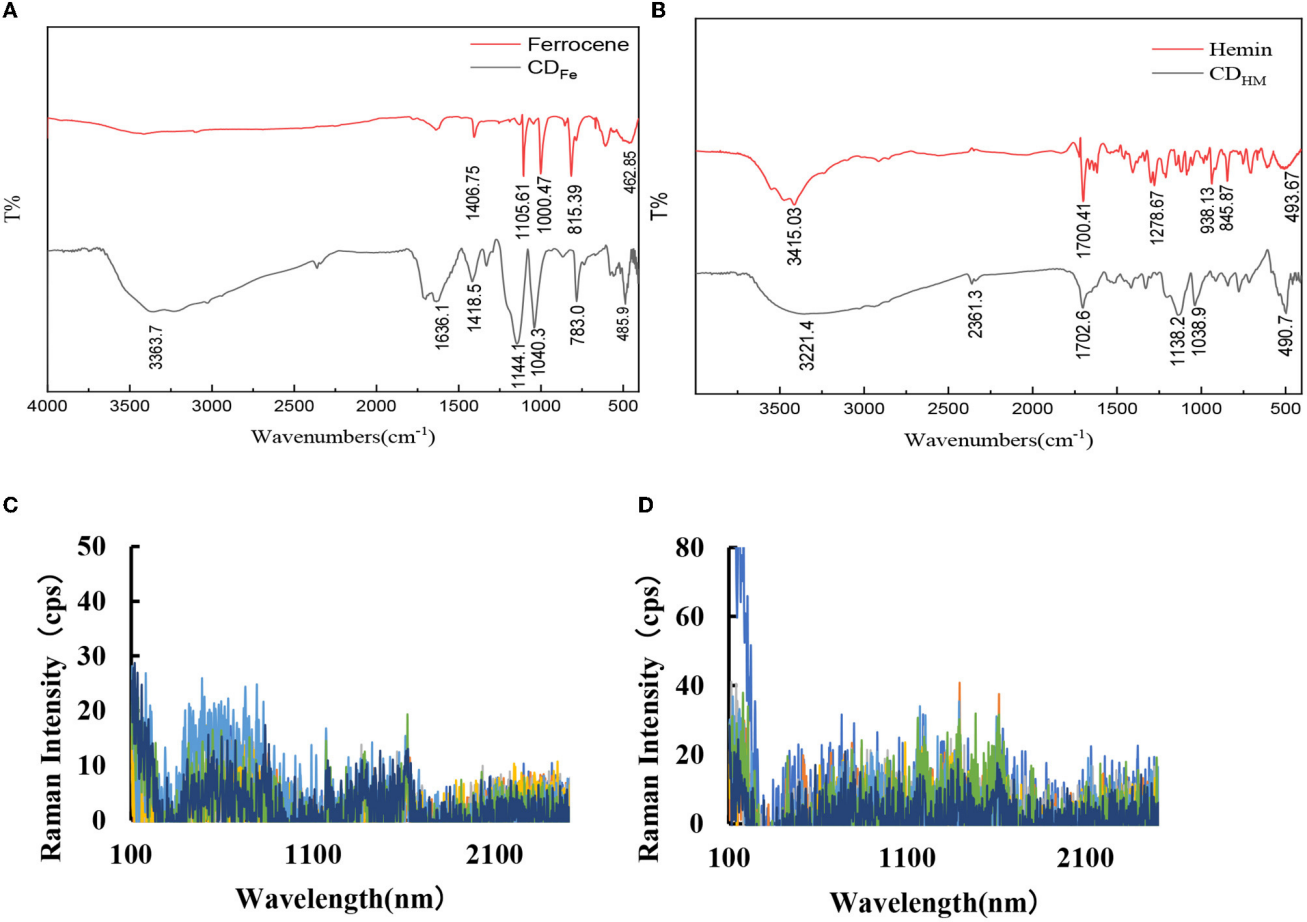
Infrared spectra and SERS spectra of CD_Fe_ and CD_HM_. **(A)** Infrared spectrum of CD_Fe_. **(B)** Infrared spectrum of CD_HM_. **(C)** SERS spectrum: 8.0 × 10^−5^ mol/L AgNPs + CD_Fe_ (0, 16, 32, 48, 60, 96, and 128 mg/L). **(D)** SERS spectrum: 8.0 × 10^−5^ mol/L AgNPs + CD_HM_ (0, 32, 48, 64, 96, 128, and 160 mg/L).

Infrared spectroscopy (FTIR) shows that there are 7 characteristic peaks of CD_Fe_ (Figure 2A): 485.9 cm^−1^ is the characteristic peak of Fe–O, 783.0 cm^−1^ was caused by the C–H out-of-plane bending vibration, 1,040.3 and 1,144.1 cm^−1^ are attributed to the C–H in-plane deformation of the benzene compound, and 1,418.5 cm^−1^ is due to the tensile vibration of the C–O. The 1,636.1 cm^−1^ is caused by the vibration of the framework of the substituted benzene C = C, and the 3,363.7 cm^−1^ is caused by the tensile vibration of C–OH, which may also be caused by the crystal water in the test sample. In the figure, the peak of ferrocene at 462.85 cm^−1^ is caused by the stretching vibration of the cyclolocene, and Fe, 815.39 cm^−1^ is attributed to πC–H, 1,000.47 cm^−1^ is formed by the C–H deformation vibration on the cyclolocene, 1,105.61 cm^−1^ is caused by the reverse stretching vibration of the ring, and 1,406.75 cm^−1^ is attributed to the C = C stretching vibration on the locene ring. The Fe–O peak was formed while CD_Fe_ was formed by Fer hydrothermal method, which indicated that its structure has changed after calcination at high temperature. Moreover, CD_Fe_ has oxygen-containing groups, which indicates that it has superior hydrophilicity and stability. At the same time, the infrared spectra of CD_HM_ and its precursor Hemin are also given (Figure 2B). It can be seen from the figure that CD_HM_ peaks are at 2,361.3, 1,138.2, and 1,038.9 cm^−1^ compared to Hemin. We studied the SERS spectrum of CD_Fe_ with AgNPs as the SERS substrate, and the results showed that there was no obvious SERS peak (Figure 2C). Finally, the SERS spectra of CD_HM_ were also studied using AgNPs as the substrate. Similar to CD_Fe_, there was no obvious SERS signal (Figure 2D).

#### 3.2.4. Transmission electron microscope and x-ray diffraction of CD_Fe_

**Figure 3:**
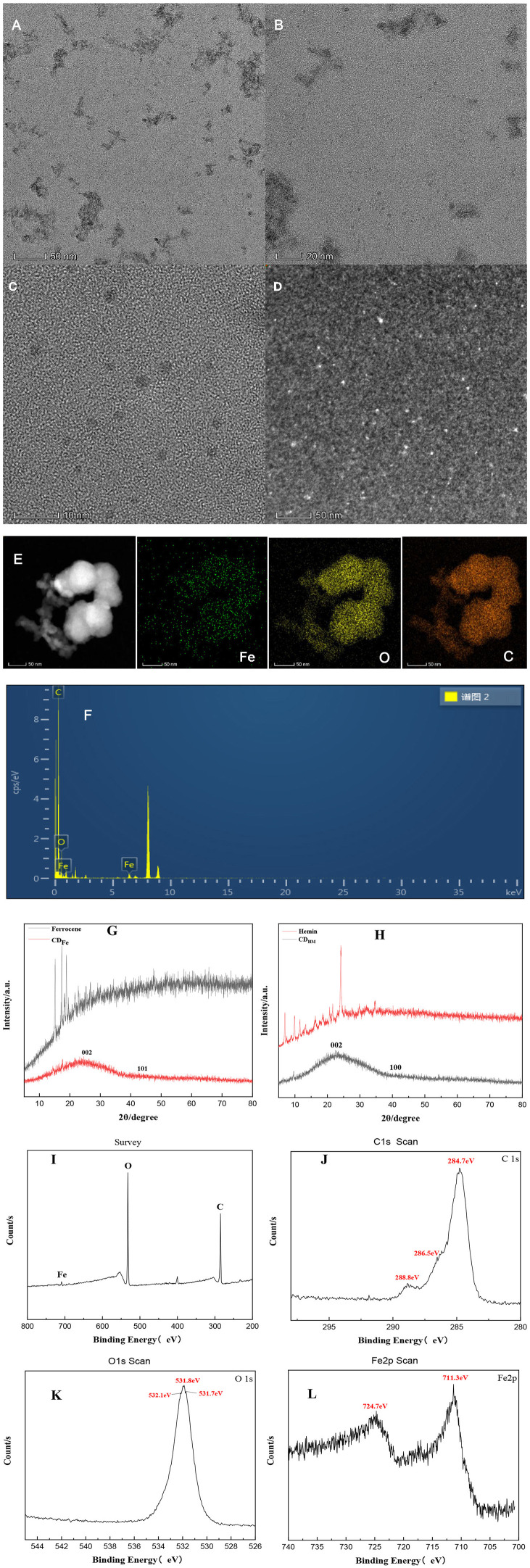
TEM, HAADF-STEM, mapping, XRD, and XPS of CD_Fe_. **(A–C)** TEM OF CD_Fe_. **(D)** HAADF-STEM of CD_Fe_. **(E)** Mapping of CD_Fe_. **(F)** EDS of CD_Fe_. **(G)** XRD of Fer and CD_Fe_. **(H)** XRD of Hemin and CD_HM_. **(I)** XPS survey spectra of CD_Fe_. **(J–L)** C1s, O1s, and Fe2p high-resolution spectrum of CD_Fe_.

Figure 3A is the TEM image taken at 50 nm resolution after the sample was diluted with ethanol after centrifugation. Figures 3B, C are the TEM images taken directly at 20 and 10 nm resolution on CD_Fe_, respectively, with the particle size ranging from 1.5 to 3.5 nm. Figure 3D is the HAADF-STEM image of CD_Fe_. Figure 3E shows that Fe is uniformly distributed on the carbon quantum dots. CD_Fe_ also contains C and O elements, but the content of Fe is relatively small. The results of the combined XRD analysis show that Fe can bind not only to cyclopentadiene but also to O, that is, Fe is anchored on the surface of carbon quantum dots in the form of Fe–O and Fer–Fe bonds. Figure 3F is the EDS of CD_Fe_. The XRD of CD_Fe_ was studied. The XRD analysis results show (Figure 3G) that the CD_Fe_ produced by the Fer hydrothermal method has a characteristic peak at about 25° and 40–45°, respectively, which are the peaks of graphitic carbon (002) and (101), indicating that CD_Fe_ has the amorphous carbon structure. At the same time, the phases of iron and iron compounds are not seen in XRD, which indicates that there are no obvious Fe particles in CD_Fe_. Fe may be combined with cyclopentadiene and O. Similarly, in the XRD study of CD_HM_ and Hemin, CD_HM_ has the same amorphous carbon structure as CD_Fe_, but there is a peak around 10° (Figure 3H). Figure 3I is the full XPS spectrum of CD_Fe_. The characteristic peaks at 284.7, 531.8, and 711.3 eV correspond to C1s, O1s, and Fe2p, respectively. It can be seen from the figure that CD_Fe_ contains three elements: carbon, oxygen, and iron. Their relative atomic percentage contents are 69.42, 28.31, and 2.27%, respectively. Figure 3J is the high-resolution energy spectrum of C1s of CD_Fe_. Three element characteristic peaks are fitted at 284.7, 286.5, and 288.8 eV, corresponding to the C–C/C–H, C–O, and C=O bonds, respectively. Figure 3K is the high-resolution energy spectrum of O1s of CD_Fe_, the characteristic peaks of the elements at 531.7, 531.8, and 532.1 eV correspond to Fe–O, –OH, and C–O bonds, respectively; Figure 3L is the high-resolution energy spectrum of Fe2p of CD_Fe_, the peak at 711.3 eV is Fe2p_3/2_, and the peak at 724.7 eV corresponds to Fe2p_1/2_. The characteristic peaks at these two peaks can indicate that Fe in CD_Fe_ exists in the chemical state of +3 valence and in the oxidized state. According to the infrared spectrum and XPS analysis of CD_Fe_, the CDs were successfully doped with iron.

#### 3.2.5. Stability of nanosol

**Figure 4:**
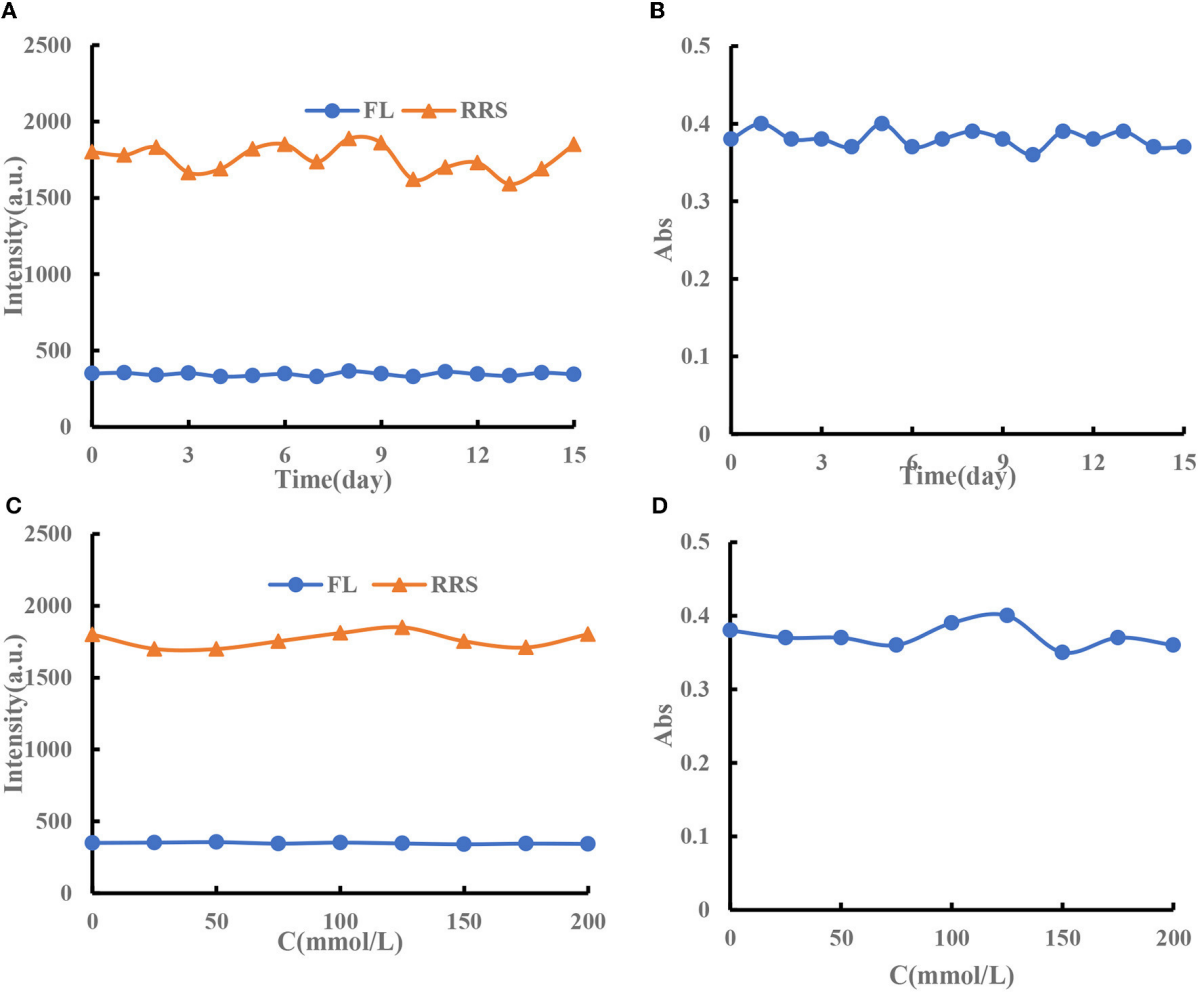
**(A)** FL and RRS signal changes of 96 mg/L CD_Fe_ over time. **(B)** Abs signal changes of 96 mg/L CD_Fe_ over time. **(C)** FL and RRS signal changes of 6 mg/L CD_Fe_ in different concentrations of NaCl solution. **(D)** Abs signal changes of 6 mg/L CD_Fe_ in different concentrations of NaCl solution.

The prepared CD_Fe_ and CD_HM_ were stored in a refrigerator at 4°C. The stability of CD_Fe_ was examined by fluorescence (FL), RRS, and UV. It was found that the signals of CD_Fe_ changed a little within 15 days, with RSDs of 3.2, 5.2, and 2.9% (Figures 4A, B). The test stated that the prepared CD_Fe_ had good stability over time. In addition, the stability of CD_Fe_ in different concentrations of NaCl solution was investigated. The results showed that the FL, RRS, and Abs signals of CD_Fe_ were stable in different concentrations of NaCl solution (Figures 4C, D). The experimental results show that the preparation method has good stability. Under naked eye observation, there will be no visible precipitation within 10 days, and its stability is good. Although CD_HM_ can make molecular spectra, after reacting at 180°C for 2 h, cooling and standing for half an hour will produce obvious precipitation. It is suspected that it may be carbonized for too long, then try to burn at 30, 60, and 90 min, but all of them were precipitated, thus, the experiment was continued without using this catalyst.

### 3.3. SERS spectra of nanocatalysis analysis system

**Figure 5:**
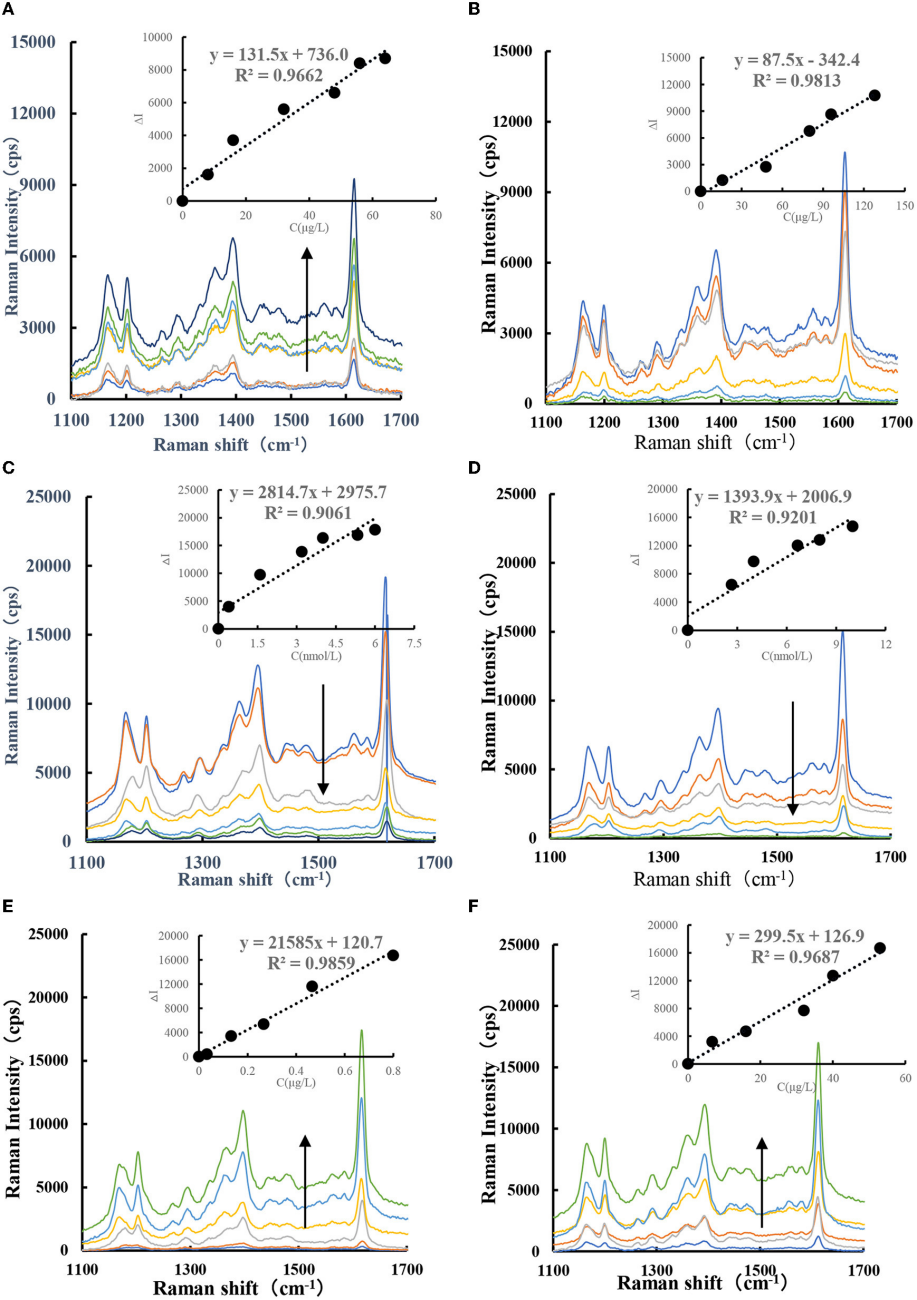
SERS spectra of CD_Fe_-GX-HAuCl_4_ catalytic/inhibition/analysis system. **(A)** (0, 8, 16, 24, 32, 48, 56, and 64 μg/L) CD_Fe_-53.3 μg/ml, HAuCl_4_-1.2 mmol/L, HCl-3.48 mmol/L, GX−6.67 × 10^−7^ mol/L VBB. **(B)** (0, 16, 48, 80, 96, and 128 μg/L) CD_HM_-53.3 μg/ml, HAuCl_4_-1.2 mmol/L, HCl-3.48 mmol/L, GX-6.67 × 10^−7^ mol/L VBB. **(C)** (0, 0.4 1.6, 3.2, 4, 5.3, and 6 nmol/L) Apt_CBZ_-48 μg/L, CD_Fe_-53.3 μg/ml, HAuCl_4_-1.2 mmol/L, HCl-3.48 mmol/L, GX-6.67 × 10^−7^ mol/L. **(D)** (0, 2.6, 4, 6.7, 8, and 10 nmol/L) Apt_PF_-48 μg/L, CD_Fe_-53.3 μg/ml, HAuCl_4_-1.2 mmol/L, HCl-3.48 mmol/L, GX-6.67 × 10-7 mol/L VBB. **(E)** (0, 0.03, 0.13, 0.26, 0.46, and 0.8 μg/L) CBZ + 4 nmol/L, Apt_CBZ_-48 μg/L, CD_Fe_-53.3 μg/ml, HAuCl_4_-1.2 mmol/L, HCl-3.48 mmol/L, GX-6.67 × 10^−7^ mol/L VBB. **(F)** (0, 6.7, 16, 32, 40, and 53 μg/L) PF + 8 nmol/L, Apt_PF_-48 μg/L, CD_Fe_-53.3 μg/ml, HAuCl_4_-1.2 mmol/L, HCl-3.48 mmol/L, GX-6.67 × 10^−7^ mol/L VBB.

With AuNPs as the SERS substrate, adding VBB molecules can generate strong SERS signals. When CD_Fe_/CD_HM_ nanosol is used as a catalyst, GX-HAuCl_4_ reacts to generate a large number of AuNPs. After adding VBB molecular probes, the system generated three SERS peaks at 1,202, 1,396, and 1,613 cm^−1^. The SERS peaks at 1,202 and 1,396 cm^−1^ are caused by the C–H bond deformation in the saturated CH = CH bond. The SERS peak at 1,613 cm^−1^ is caused by the strong stretching vibration of the C = C bond in the dicyclopentadiene framework. The SERS signal at 1,613 cm^−1^ is the strongest, and the SERS intensity gradually increases with the CD_Fe_/CD_HM_ concentration (Figures 5A, B). As can be seen from the slope of SERS spectrum of CD_Fe_/CD_HM_ catalytic system, the catalytic effect of CD_Fe_ is significantly stronger than that of CD_HM_ system. Afterward, CD_Fe_ was used as a catalyst to catalyze the GX-HAuCl_4_ system at 85°C in a water bath. The results showed that Au^3+^ could be reduced to AuNPs. After adding Apt to the GX-HAuCl_4_ catalytic system, the SERS signal at 1,613 cm^−1^ of the system gradually decreased with the increase in Apt concentration (Figures 5C, D). For the CD_Fe_-Apt-HAuCl_4_-GX reaction, when the target molecule CBZ/PF was added, they would specifically bind to Apt, thereby releasing CD_Fe_ to restore its catalytic activity and releasing more CD_Fe_ when the concentration of the target molecules was increased, more AuNPs were generated. In addition, the signal intensity of the SERS spectrum increased linearly with the concentration of the target (Figures 5E, F) in a certain linear range when VBB was added.

### 3.4. RRS spectra

**Figure 6:**
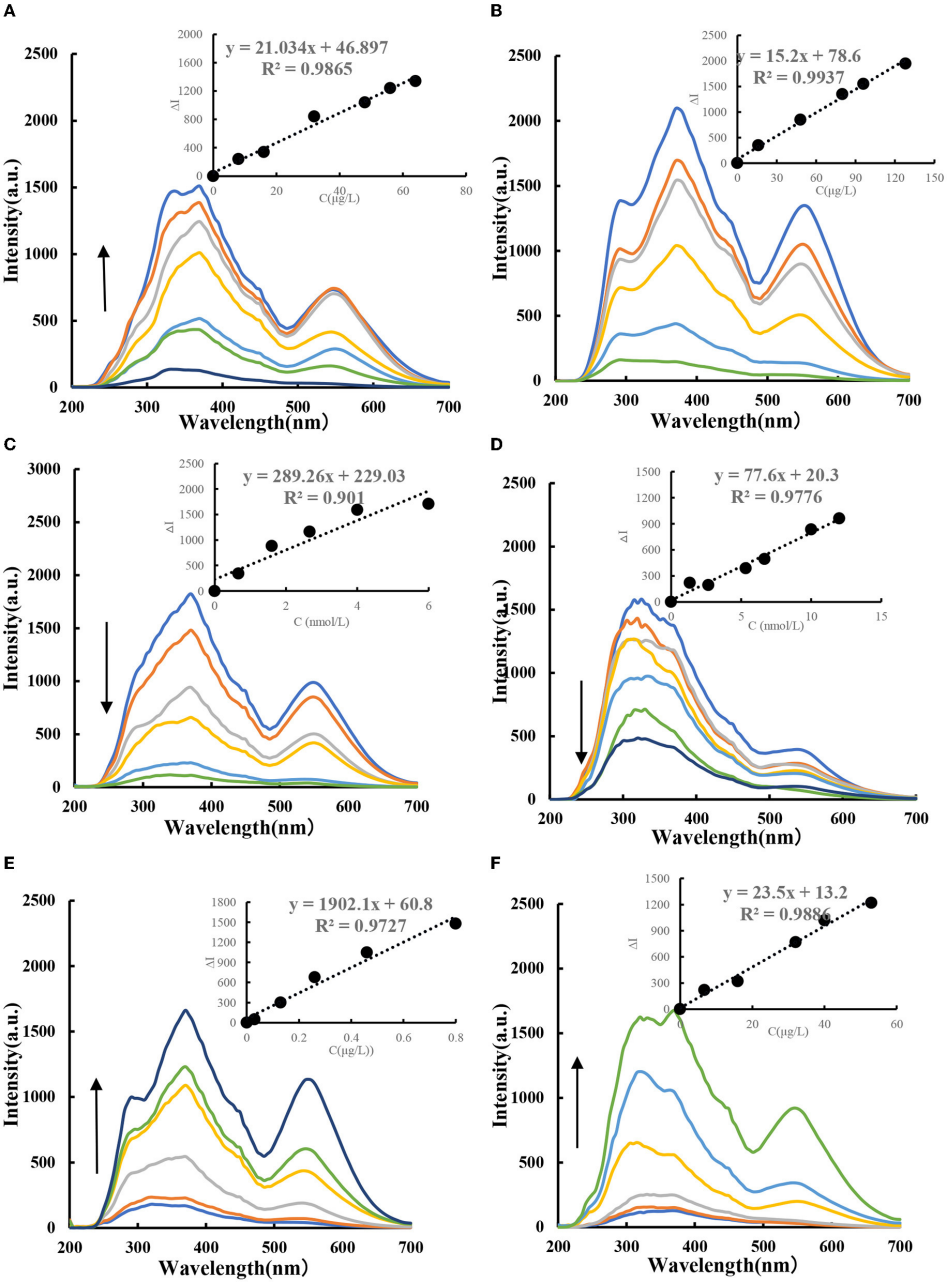
RRS spectra of CD_Fe_-GX-HAuCl_4_ catalytic/inhibition/analysis system. **(A)** (0, 8, 16, 24, 32, 48, 56, and 64 μg/L) CD_Fe_-53.3 μg/ml, HAuCl_4_-1.2 mmol/L, HCl-3.48 mmol/L GX. **(B)** (0, 16, 48, 80, 96, and 128 μg/L) CD_HM_-53.3 μg/ml, HAuCl_4_-1.2 mmol/L, HCl-3.48 mmol/L GX. **(C)** (0, 0.67, 1.6, 2.67, 4, and 6 nmol/L) Apt_CBZ_-48 μg/L, CD_Fe_-53.3 μg/ml, HAuCl_4_-1.2 mmol/L, HCl-3.48 mmol/L GX. **(D)** (0, 1.3, 2.6, 5.3, 6.7, 10, and 12 nmol/L) Apt_PF_-48 μg/L, CD_Fe_-53.3 μg/ml, HAuCl_4_-1.2 mmol/L, HCl-3.48 mmol/L GX. **(E)** (0, 0.03, 0.13, 0.26, 0.46, and 0.8 μg/L) CBZ-4.6 nmol/L, Apt_CBZ_-48 μg/L, CD_Fe_-53.3 μg/ml, HAuCl_4_-1.2 mmol/L, HCl-3.48 mmol/L GX. **(F)** (0, 6.7, 16, 32, 40, and 53 μg/L) PF-12 nmol/L, Apt_PF_-48 μg/L, CD_Fe_-53.3 μg/ml, HAuCl_4_-1.2 mmol/L, HCl-3.48 mmol/L GX.

**Figure 7:**
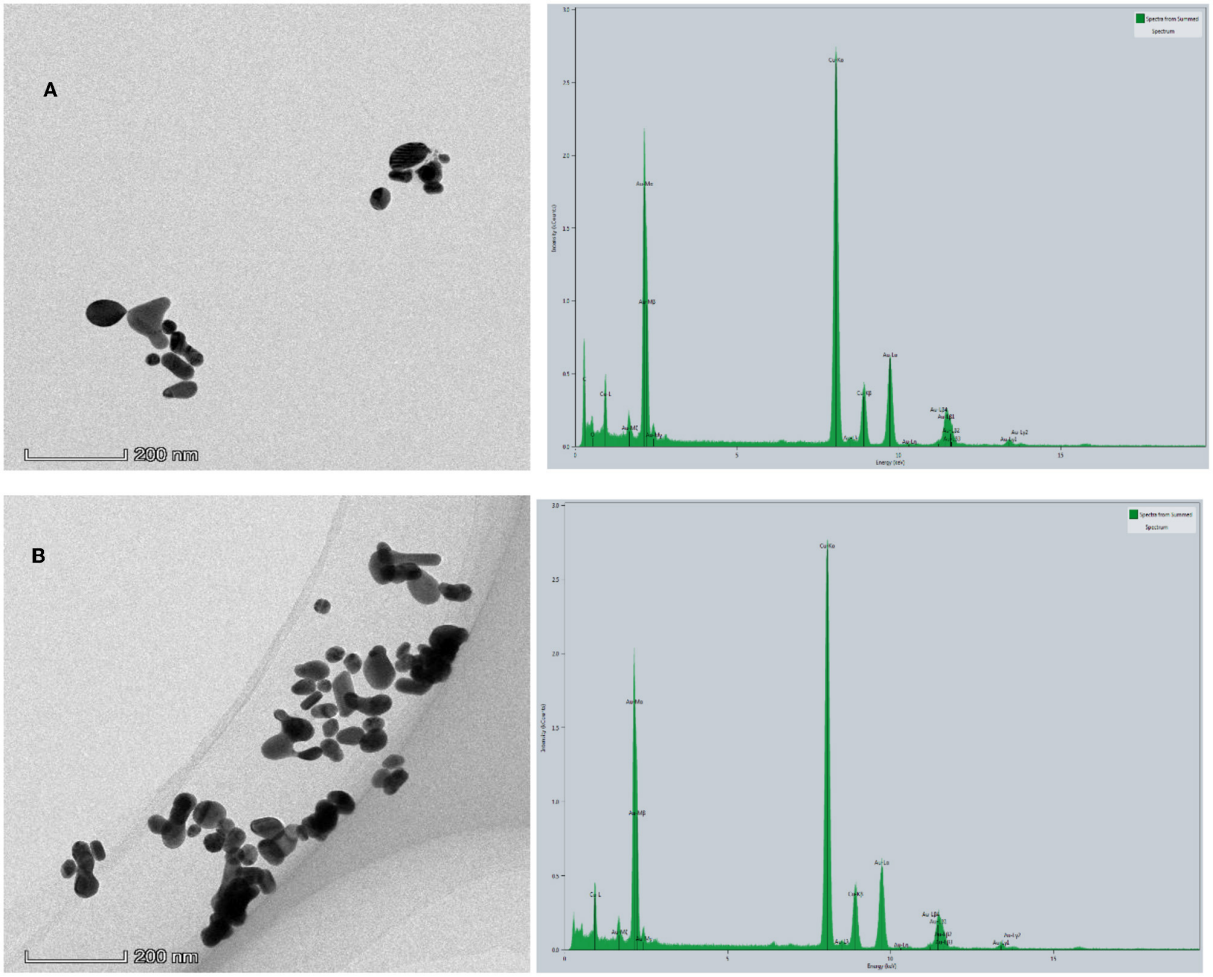
TEM and EDS of the analytical system. **(A)** 1.33 nmol/L Apt_CBZ_ + 48 μg/L CD_Fe_ + 53.3 μg/ml, HAuCl_4_ + 1.2 mmol/L, HCl + 3.48 mmol/L GX. **(B)** A + 0.08 μg/L CBZ.

The HAuCl_4_-GX-HCl-CD_Fe_ catalytic system has three spectral peaks at 280, 370, and 550 nm. As the concentration of CD_Fe_ increases, the concentration of AuNPs increases gradually and the RRS peak at 370 nm is the largest (Figure 6A). Figure 6B shows the RRS spectrum of CD_HM_ as a catalyst. It was found in the experiment that the RRS signal decreased with the addition of Apt (Figures 7C, D). For the CD_Fe_-Apt-HAuCl_4_-GX reaction, when the target molecule CBZ/PF was added, the ligand-specific binding would occur and the CD_Fe_ would be released so that the reaction system restored its catalytic activity. Within a certain linear range, as the number of target molecules increased, the signal strength of RRS would also increase (Figures 6E, F). The results showed that the sensitivity of the reaction system to CBZ was very high and the linearity was relatively good.

### 3.5. TEM of the analytical system

The reaction solution of the system was obtained according to the experimental method of Section Experimental method, and 10 μl solution was dropped on the surface of the ultra-thin carbon film three times to obtain TEM of the system. Figure 7A shows that the catalytic activity of CD_Fe_ is inhibited when CBZ is not added, and the system produces very few AuNPs, with a particle size between 20 and 100 nm and good dispersibility. After adding CBZ, CD_Fe_ was released, its catalytic activity was restored, and the number of AuNPs generated increased, with a particle size between 25 and 150 nm and aggregation (Figure 7B). There are very strong Au-M (Au-Mα, Au-Mβ, Au-Mγ) characteristic peaks signal around 2.131 keV in the energy spectrum and a relatively strong Au-Lα peak around 9.712 keV. There are also signals of Au-Lβ (Au-Lβ_1_, Au-Lβ_2_, Au-Lβ_3_, Au-Lβ_4_) characteristic peaks around 11.440 and 11.583 keV, which proved that AuNPs were generated in this system.

### 3.6. CD_Fe_ catalysis and aptamer inhibition

**Figure 8:**
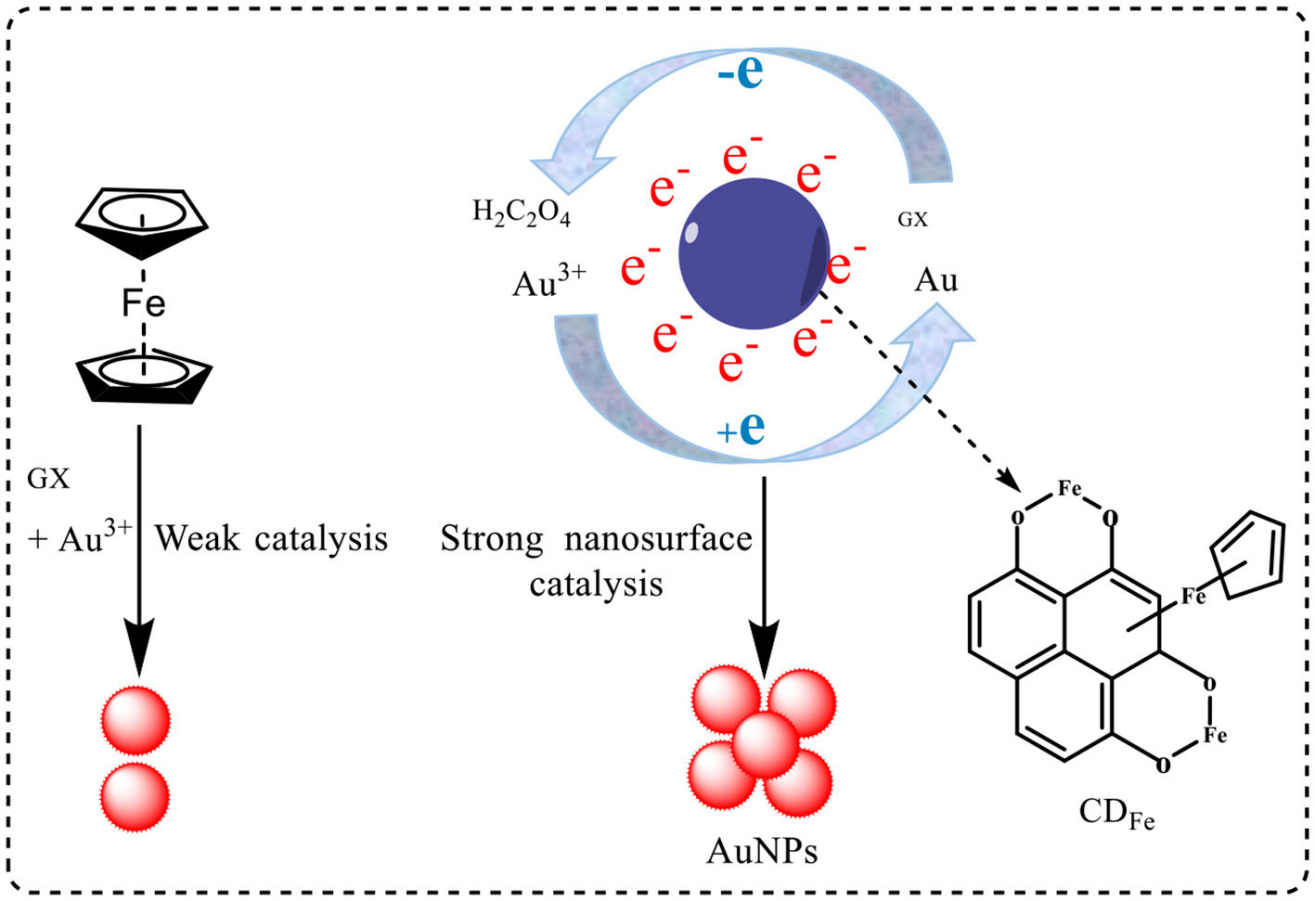
Mechanism of CD_Fe_ nanocatalytic enhancement.

Under experimental conditions, both CD_Fe_ and CD_HM_ could catalyze the reduction of HAuCl_4_ by GX to produce AuNPs. In a certain concentration range, with the increase in catalyst concentration, the number of AuNPs was generated, and the SERS and RRS signals of the system had a linear relationship with the concentration of the catalyst. According to the elementary reaction principle, Au (III) was reduced to Au(I) and Au atoms were aggregated into AuNPs. The GX was oxidized to oxalate and CO_2_, in the presence of highly efficient nanocatalyst of CD_Fe_. After the addition of the Apt, it was adsorbed on the surface of the nanocatalyst by intermolecular forces, thereby inhibiting its catalytic activity and the SERS and RRS signals of the system showed a linear decrease trend with the increase in the amount of Apt in a certain concentration range. Under the conditions of high temperature and high pressure, ferrocene was carbonized to form CD_Fe_. Fe through –Fe–O– and –Fe– bond dispersion anchor on the surface of CD to form a CD_Fe_ catalyst, the surface of CD_Fe_ catalyst was rich in π electrons and Fe metal electrons, both of which could promote electron transfer. HAuCl_4_ and glyoxal could be also adsorbed on the surface of CD_Fe_ to promote the transfer of redox electrons. Thus, AuNP reaction was enhanced greatly (Figure 8). The main reactions were as follows:


(1) $$\begin{array}{l} \text{Au}\left(\text{III}\right)+\text{GX}=\text{Au}\left(\text{I}\right)+\text{oxalate} \end{array}$$

(2) $$\begin{array}{l} \text{Au}\left(\text{III}\right)+\text{oxalate}=\text{Au}\left(\text{I}\right)+\text{oxalate} \end{array}$$

(3) $$\begin{array}{l} \text{Au}\left(\text{I}\right)+\text{GX}=\text{Au}+\text{oxalate} \end{array}$$

(4) $$\begin{array}{l} \text{Au}\left(\text{I}\right)+\text{oxalate}=\text{Au}+\text{C}{\text{O}}_{2} \end{array}$$

(5) $$\begin{array}{l} \text{nAu}=\text{AuNP} \end{array}$$

### 3.7. Selection of analysis conditions

With the increase in the amount of CD_Fe_, the number of AuNPs generated by the reaction increased, and the RRS signal of the system was enhanced continuously. When the amount of CD_Fe_ was 0.048 mg/L, the ΔI of the system reached the maximum. Thus, the dosage of CD_Fe_ was 0.048 mg/L (Supplementary Figure 6A). Apt could interact with CD_Fe_ to form CD_Fe_-Apt on its surface, thereby weakening its catalytic effect. When CBZ was added, Apt combined with CBZ, causing CD_Fe_ to be exposed again and restore its catalytic activity, and the catalytic reaction proceeded smoothly. In excess, Apt wrapped on the surface of CD_Fe_ and still inhibited the catalytic reaction. In addition, the results showed that when the dosage of Apt was 4.6 nmol/L (Supplementary Figure 6B), the system had the best response effect. When the amount of GX was continuously increased, due to the rapid generation of AuNPs, the aggregation of AuNPs would lead to a decrease in ΔI in the system. The concentration of GX was 3.48 mmol/L (Supplementary Figure 6C). When the dosage of HAuCl_4_ was continuously increased, the accumulation of AuNPs occurred due to the rapid formation of AuNPs. At the same time, the blank also reacted, and the ΔI of the system decreases. Thus, when the dosage of HAuCl_4_ was 0.053 mg/ml, the ΔI of the system reached the maximum (Supplementary Figure 6D). When there was no HCl in the system, the rate of AuNP generation was too fast, the AuNPs were aggregated, and the ΔI of the system was low. As the concentration of HCl increased, the rate of AuNPs generation gradually slowed down, the amount of AuNPs produced by the reaction also increased, and the RRS signal of the system continued to increase. When the amount of HCl was continuously increased, the production of AuNP was inhibited, and the ΔI of the system decreases instead. Therefore, the amount of HCl used in this experiment was 1.2 mmol/L (Supplementary Figure 6E). The temperature had a great influence on the reaction system. The higher the temperature, the faster the reaction speed of the system. At 60°C, the reaction did not occur, but when the temperature was too high, the reaction speed was too fast and the system was unstable. When the reaction temperature was 80°C, the ΔI of the system reached the maximum. Therefore, the reaction temperature was selected as 80°C in the experiment (Supplementary Figure 6F). At the reaction temperature of 80°C, the reaction of the system gradually accelerated with time and reached the highest value at 35 min. After 35 min, the reaction occurred completely, but the blank also began to react. Therefore, the reaction time of this experiment was selected as 35 min (Supplementary Figure 6G).

### 3.8. Working curve

Under the selected optimal test conditions, the linear regression equations of all the methods for detecting the analytes presented in this article are shown in Table 1 with the concentration of the analyte and the corresponding RRS value ΔI_370nm_ and the SERS value ΔI_1,613cm−1_. It could be seen from the table that the SERS method was better and its sensitivity was higher. The RRS method was simpler than the SERS without molecular probes. Compared with the reported analytical methods for the determination of CBZ (Table 2), this new SERS method was one of the most sensitive methods.

**Table 1:** Comparison of analysis characteristics of the discattering method.

**Analyte**	**Method**	**LR (μg/L)**	**Linear equation**	**Correlation (*R^2^*)**	**DL (μg/L)**
CBZ	SERS	0.03–0.8	ΔI_1613cm − 1_ = 21585C + 120.7	0.9859	0.003
PF		6.7–53.0	ΔI_1613cm − 1_ = 299.5C+126.9	0.9687	0.4
CBZ	RRS	0.03–0.8	ΔI_370nm_ = 1902.1C + 60.8	0.9727	0.01
PF		6.7–53.0	ΔI_370nm_ **=** 23.51C + 13.1	0.9886	0.06

**Table 2:** Comparison of some reported spectral methods for CBZ.

**Method**	**Principle**	**Linear range**	**Detection limit**	**Notes**	**References**
SERS	Nanocellulose can be used as a framework for loading AgNPs and serve as a substrate for SERS. The fabrication of silver-nanocellulose composites was synthesized. A commonly used CBZ was used as a SERS probe to evaluate the properties of NCF-Ag.	10^−4^-10^−7^ M	1.0 × 10^−8^ M	Low-cost, environmentally friendly	(25)
Colorimetric method	Determination of CBZ by specific Apts of unlabeled CBZ, AuNPs, and cationic polymer poly-diallyldimethylammonium chloride	2.2–500 nM	2.2 nM	Simple pretreatment, short duration, and obvious results	(26)
Electrochemical	Platinum-doped nickel cobaltite nanograss/screen-printed electrode (SPE) on the CBZ oxidation signal.	0.03–140 μM	0.005 μM	Good anti-interference ability, selectivity, and stability	(27)
Electrochemical sensor	Combining C-ZIF67@Ni with MIP, it was developed a high-sensitivity and high-selectivity CBZ electrochemical sensor.	4 × 10^−13^-1 × 10^−9^ M	1.35 × 10^−13^ M	Favorable sensitivity, selectivity, stability, and reliability.	(28)
SERS/RRS	Coupled CD_Fe_ catalytic reaction and the specific reaction of Apt_CBZ_-CBZ. SERS/RRS signal increased with the increase of CBZ concentration.	0.03–0.8 μg/L	0.003 μg/L; 0.01 μg/L	Good selectivity, high sensitivity, cheap	This study

### 3.9. Influence of coexisting substances

The influence of coexisting substances on the determination of 0.2 μg/L CBZ was investigated according to the test method, and it was found that the influence of OTC, PF, triadimefon, isocarbophos, benzoic acid, Mg^2+^, Ca^2+^, Cr^2+^, Ba^2+^, ${\text{HCO}}_{3}^{2-}$, ${\text{NH}}_{4}^{-}$, and Al^3+^ were 100 times. Notably, 50 times were Fe^3+^, Zn^2+^, Cu^2+^, ${\text{HPO}}_{4}^{2-}$, ${\text{P}}_{2}{\text{O}}_{7}^{4-}$, and BSA, 10 times of ${\text{CO}}_{3}^{2-}$, and HSA had no interference (Supplementary Table 1). The results indicate that this system has high selectivity.

### 3.10. Analysis application

The tea sample weighing 5.0 g was put into a 100-ml conical flask, then 50 ml methylene chloride was added. After vortex oscillation for 3 min, it was pumped and filtered in a sand core funnel paved with Celite 545, evaporated and concentrated at 45°C, and blown dry with nitrogen. A 0.2 g C_18_ was added and fixed to volume with 1 ml ethanol, whirled for 2 min, and filtrated by 0.22 μM membrane filtration to obtain the sample solution. The CBZ was measured according to the experimental method. The measurement results are shown in Table 3, and the SERS results are in agreement with that of the HPLS method. The recovery rate was 92.0–109%, with a relative standard deviation (RSD) of 5.4–8.4%. The results indicated the SERS assay was accurate and reliable. According to the National Food Safety Standard maximum residue limits of pesticides in foods (GB 2763-2021), the maximum content of carbendazim in apples is 5 mg/kg. The CBZ content in industrial samples did not exceed the standard.

**Table 3:** SERS determination of CBZ in tea.

**Tea**	**Average (mg/kg, *n* = 5)**	**Added (mg/kg)**	**Measured (mg/kg)**	**Recovery (%)**	**RSD (%)**	**HPLC (mg/kg)**
1	0.250	0.500	0.760	102	5.9	0.218
2	0.198	0.500	0.720	104	8.4	0.220
3	0.156	0.500	0.700	109	5.4	0.160
4	1.06	0.500	1.52	92.0	7.0	0.980
5	0.470	0.500	0.934	92.8	5.9	0.464

## 4. Conclusion

A new CD_Fe_ was prepared and characterized in detail. A new SERS/RRS dimode indicator reaction of CD_Fe_-Au (III)-GX was constructed. It was coupled with the Apt reaction to construct a dimode Apt biosensor analysis platform for detecting CBZ. In addition, this new strategy can be used to detect PF. There are some limitations to this study, including limited Apt species and complex combination of catalyst and Apt, and further studies on looking for nanoprobes that can be used as both catalysts and specific recognition functions to expand the application of this platform.

## Data availability statement

The original contributions presented in the study are included in the article/Supplementary material, further inquiries can be directed to the corresponding authors.

## Author contributions

HB and RZ: software, visualization, writing original draft, investigation, and formal analysis. CL and AL: visualization, conception, reviewing and editing, methodology, validation, formal analysis, and supervision. All authors contributed to the article and approved the submitted version.
