# Supplementary material for: Nanogold sol plasmon discattering assay for trace carbendazim in tea coupled aptamer with Au3+-glyoxal-carbon dot nanocatalytic reaction
Source: Front Nutr. 2023 Mar 6;10:1122876. doi: 10.3389/fnut.2023.1122876 (PMC10025530; doi:10.3389/fnut.2023.1122876)
Supplement: Supplementary file 1 [file Data_Sheet_1.docx]

**Nanogold sol plasmon di-scattering assay for trace carbendazim in tea coupled aptamer with Au^3+^-glyoxal-carbon dot nanocatalytic reaction**

Hongyan Bai ^1,2^, Ran Zhang ^1,2^, Chongning Li^1,2*^, Aihui Liang ^2*^

^1^ School of Public Health, Guilin Medical University, Guilin 541199, China; ^2^Guangxi Key Laboratory of Environmental Pollution Control Theory and Technology, Guilin 541006, China.

|  |  |
| --- | --- |
|  |  |

**FIGURE S1.** RRS Spectra of CD_Fe_-GX-HCl-HAuCl_4_ Catalytic System at Different Time. (A) CD_Fe_ (80 min)-53.3 μg/mL HAuCl_4_-1.2 mmol/L HCl-3.48 mmol/L GX, a-h curves represent 0, 8, 16, 32, 40, 48, 60 and 72 μg/L CD_Fe_. (B) CD_Fe_ (100 min)-53.3 μg/mL HAuCl_4_-1.2 mmol/L HCl-3.48 mmol/L GX, a-g curves represent 0, 16, 32, 40, 48, 72 and 80 μg/L CD_Fe_. (C) CD_Fe_ (120 min)-53.3 μg/mL HAuCl_4_-1.2 mmol/L HCl-3.48 mmol/L GX, a-f curves represent 0, 8, 16, 32, 64 and 80 μg/L CD_Fe_. (D) CD_Fe_ (150 min)-53.3 μg/mL HAuCl_4_-1.2 mmol/L HCl-3.48 mmol/L GX, a-f curves represent 0, 8, 16, 32, 60 and 72 μg/L CD_Fe_.

|  |  |
| --- | --- |
|  |  |

**FIGURE S2.** RRS Spectra of CD_Fe_-GX-HCl-HAuCl_4_ Catalytic System at Different Temperatures. (A) CD_Fe_ (140℃)-53.3 μg/mL HAuCl_4_-1.2 mmol/L HCl-3.48 mmol/L GX, a-f curves represent 0, 0.8, 1.6, 2.4, 3 and 3.6 mg/L CD_Fe。_ (B) CD_Fe_ (160℃)-53.3 μg/mL HAuCl_4_-1.2 mmol/L HCl-3.48 mmol/LGX, a-f curves represent 0, 0.8, 1.6, 2, 2.4 and 3.6 mg/L CD_Fe。_(C) CD_Fe_ (180℃)-53.3 μg/mL HAuCl_4_-1.2 mmol/L HCl-3.48 mmol/L GX, a-f curves represent 0, 8, 16, 32, 64 and 80 μg/L CD_Fe。_(D) CD_Fe_ (200℃)-53.3 μg/mL HAuCl_4_-1.2 mmol/L HCl-3.48 mmol/L GX, a-f curves represent 0, 16, 24, 32, 72 and 96 μg/L CD_Fe。_

|  |  |
| --- | --- |
|  |  |
|  |  |

**FIGURE S3.** RRS Spectra of CD_Fe_-GX-HCl-HAuCl_4_ Catalytic System with Different Fer Dosages. (A)CD_Fe_ (0.012 g)-53.3 μg/mL HAuCl_4_-1.2 mmol/L HCl-3.48 mmol/L GX, a-f curves represent 0, 5.33, 10.67, 32, 40 and 55 μg/L CD_Fe_. (B) CD_Fe_ (0.018 g)-53.3 μg/mL HAuCl_4_-1.2 mmol/L HCl-3.48 mmol/L GX, a-f curves represent 0, 8, 16, 32, 64 and 80 μg/L CD_Fe_. (C) CD_Fe_ (0.024 g)-53.3 μg/mL HAuCl_4_-1.2 mmol/L HCl-3.48 mmol/L GX, a-f curves represent 0, 10.67, 42.67, 53.3, 85.3 and 106.67 μg/L CD_Fe_. (D) CD_Fe_ (0.03 g)-53.3 μg/mL HAuCl_4_-1.2 mmol/L HCl-3.48 mmol/L GX, a-f curves represent 0, 26.67, 53.3, 66.67, 106.67 and 120 μg/L CD_Fe_. (E) CD_Fe_ (0.045 g)-53.3 μg/mL HAuCl_4_-1.2 mmol/L HCl-3.48 mmol/L GX, a-g curves represent 0, 20, 40, 100, 120, 160 and 180 μg/L CD_Fe_.

|  |  |
| --- | --- |
|  |  |

**FIGURE S4.** Effect of reaction time/reaction temperature/Fer dosage on k. (A) 0.018 g Fer-x h-180℃, (B) 0.018 g Fer-2 h-x℃, (C) x g Fer-2 h-180℃.

| **A** |  |
| --- | --- |
|  |  |
|  |  |
|  | 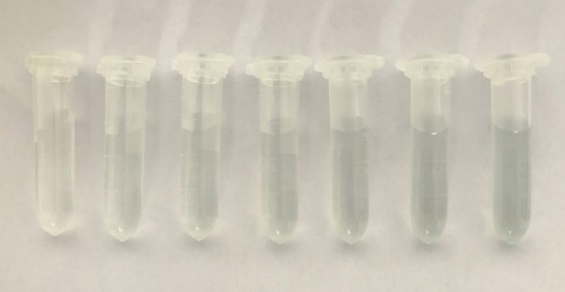 |
|  | 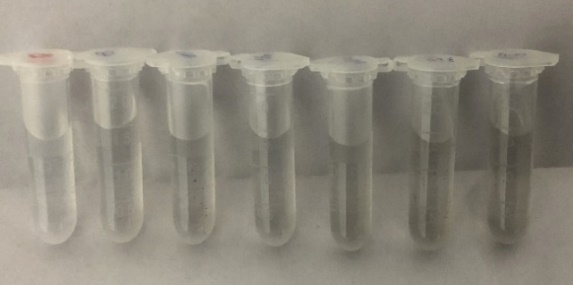 |

**FIGURE S5.** Fluorescence/RRS/Abs spectrum of CD_Fe_/CD_HM_. (**A)** The fluorescence spectrum of CD_Fe_ under different excitation wavelengths, 128 mg/L CD_Fe_. (**B)** CD_Fe_ fluorescence spectrum, the a-g curves represent 0, 16, 32, 60, 72, 96 and 128 mg/L CD_Fe_, respectively. (**C)** CD_HM_ fluorescence spectrum, a-g curves represent 0, 8, 16, 60, 32, 48, 64 and 96 mg/L CD_HM_ respectively. (**D**) CD_Fe_ RRS spectrum, a-f curves represent 0, 24 40, 72, 80 and 96 mg/L CD_Fe_ respectively. (**E)** CD_HM_ RRS spectrum, a-h curves represent 0, 8, 16, 24, 48, 64, 96 and 128 mg/L CD_HM_. (**F)** CD_Fe_ Abs spectrum, a-g curves represent 0, 16, 32, 48, 60, 96 and 128 mg/L CD_Fe_. (**G)** CD_HM_ Abs spectrum, a-g curves represent 0, 32, 48, 64, 96, 128 and 160 mg/L CD_HM_ respectively.

**FIGURE S6.** Selection of conditions. (**A)** The influence of CD_Fe_ concentration on the ∆I. (**B)** The influence of Apt_CBZ_ on the system ∆I. (**C)** The influence of glyoxal concentration on the ∆I of the system. (**D)** The effect of HAuCl_4_ concentration on the ∆I of the system. (**E)** The influence of the concentration of HCl on the ∆I of the system. (**F**) The influence of temperature on the ∆I of the system. (**G)** The effect of time on the ∆I of the system.

**TABLE S1**. Influence of coexisting substances on SERS determination of CBZ

| Interfering ion | Tolerate limit | Relative error (%) | Interfering ion | Tolerate limit | Relative error (%) |
| --- | --- | --- | --- | --- | --- |
| Mg^2+^ | 100 | -9.79 | Ca^2+^ | 100 | -6.81 |
| Cr^2+^ | 100 | 0.34 | Ba^2+^ | 100 | 0.03 |
| HCO_3_^2-^ | 100 | 6.96 | NH_4_^-^ | 100 | 5.0 |
| Al^3+^ | 100 | -7.43 | Cu^2+^ | 50 | -7.08 |
| HPO_4_^2--^ | 50 | 3.61 | P_2_O_7_^4-^ | 50 | -1.55 |
| Fe^3+^ | 50 | 2.83 | Zn^2+^ | 50 | 5.12 |
| BSA | 50 | 8.77 | HSA | 10 | 9.43 |
| CO_3_^2-^ | 10 | 0.66 | PF | 100 | 4.9 |
| OTC | 100 | 5.3 | triadimefon | 100 | 5.3 |
| Isocarbophos | 100 | 4.8 | Benzoic acid | 100 | 2.6 |
